# Increasing Self-Efficacy for the Management of Patients with Type 2 Diabetes Through an Advanced Practice Education Program for Primary Care Professionals

**DOI:** 10.3390/nursrep14040280

**Published:** 2024-12-05

**Authors:** Bushra Yunis, Paloma Echevarría-Pérez, Juan Jose Hernandez Morante, Isabel Morales-Moreno

**Affiliations:** 1 North District Meuhedet HMO, Tel-Aviv University, Tel-Aviv 6997801, Israel; bushrayunis@walla.com; 2 Faculty of Nursing, Universidad Católica de Murcia, Campus de Guadalupe, 30107 Murcia, Spain; pechevarria@ucam.edu (P.E.-P.); imorales@ucam.edu (I.M.-M.)

**Keywords:** primary care, advanced practice, health staff, type 2 diabetes, education, self-efficacy, knowledge

## Abstract

Background/Objectives: Previous studies have shown that primary care (PC) professionals have a low knowledge about the management of patients with type 2 diabetes, despite being one of the most common chronic diseases. The objective of this study is to analyze the impact of an educational program for health professionals on the metabolic control of their patients diagnosed with type 2 diabetes. Methods: This work follows a quasi-experimental longitudinal design following a double perspective. First, an educational intervention was conducted on primary care health professionals. Previous diabetes knowledge was evaluated on 157 PC health professionals. Those with lower knowledge scores (<3.5 out of 7) were allocated to the intervention group. An 8-week advanced education program was conducted on 77 PC health professionals. Self-efficacy and quality of care were evaluated. Second, a prospective cohort study was conducted to evaluate changes in metabolic parameters in their patients with type 2 diabetes. A total of 4099 patients with type 2 diabetes attending PC services were divided depending on the formation of health professionals. Biochemical and other clinical parameters were determined at baseline and after 12 months; the study was allocated in the Primary Health Centers of Meuhedet North District (Israel), from January 2022 to June 2023. Changes from the baseline were compared using ANOVA. Additionally, a mixed-effect model was conducted to capture variability within primary care staff and between groups of patients. Results: The education program significantly improved health staff knowledge (*p* < 0.001) and all dimensions of self-efficacy (*p* < 0.001 in all cases). These improvements were mirrored in patients’ outcomes, since those managed by health professionals attending the advanced practice education showed, after 6 months, better glucose (*p* < 0.001), HbA1c (*p* < 0.001), and eGFR (*p* = 0.006) levels. Conclusions: The advanced practice education program oriented to PC professionals was able to significantly improve their self-efficacy and perceived quality of care, which induced a significant effect on metabolic markers of patients with type 2 diabetes. Overall, the data reinforce the usefulness of advanced education programs, especially in chronic complex diseases like type 2 diabetes.

## 1. Introduction

One of the most challenging issues that health professionals are currently facing is the massive increase in type 2 diabetes prevalence, with a global prevalence of 8.5%. This situation is associated with higher direct medical costs, loss of productivity, premature mortality, and other subtle costs, such as those derived from impaired social relationships and quality of life [1]. Nevertheless, changes in the demographic characteristics of the population with diabetes, as well as changes in healthcare delivery, therapies, and access to technology may affect the management of people with diabetes [2,3]. In this context, advanced practice training for healthcare professionals in managing type 2 diabetes may significantly improve patient outcomes by enhancing metabolic control.

However, it has been described that type 2 diabetes is considered by health professionals as a trivial disease and, therefore, the management of these patients is usually taken less seriously than those patients under insulin therapy (type 1 diabetes) [4]. In fact, type 2 diabetes care is usually carried out in primary care settings, and only hospital care is conducted after severe complications. This highlights the need for specific advanced practice training for primary care professionals.

According to Weekes & Lembke, it was demonstrated that the enhanced management of type 2 diabetes in primary care settings led to greater engagement among general practitioners and advanced nurse practitioners, boosting their knowledge and confidence [5]. Fang et al. observed significant improvements in fasting blood glucose and overall patient care in primary healthcare after specific training on type 2 diabetes [6]. Furthermore, the training extends to the management of comorbid conditions like dyslipidemia, renal malfunction, and other comorbidities. Consequently, the risk of atherosclerosis and cardiovascular disease was reduced, which represents a significant reduction in morbidity and mortality in this population.

Advanced training programs for type 2 diabetes management emphasize the benefits of a multidisciplinary approach in enhancing the quality of care. For instance, training that highlights the role of a balanced diet and incorporates routine follow-ups and modifications, as reported by Rowe et al. [7], has led to notable improvements in patient outcomes. By promoting a team-based approach that includes various specialists, these programs ensure a comprehensive strategy for type 2 diabetes management.

The effectiveness of the healthcare system can also be improved by advanced practice training in type 2 diabetes, which reduces the frequency of acute exacerbations and hospital visits. This significantly lowers long-term healthcare costs and improves system efficiency. The same work by Rowe et al. demonstrated that the integration of inter-professional education and teamwork in primary care settings had led to a substantial decrease in emergency department visits [7].

In addition, training programs that integrate decision-support tools, evidence-based guidelines, and patient education not only improve healthcare delivery but also encourage patient adherence to treatment plans. Powers et al. have emphasized that such programs significantly improved participants’ knowledge and understanding of diabetes management [8], thereby further contributing to the effectiveness of training. Moreover, interaction with peers and feedback from experienced professionals during training sessions further validates their clinical approaches, boosting their confidence and effectiveness.

Despite the benefits derived from advanced training programs, there is little information regarding self-efficacy and perception of the quality of care delivered by primary care professionals. This situation is especially relevant in type 2 diabetes, where personal characteristics require customized treatment plans based on individual patient needs and comorbidities [9]. The approach developed in the present work may not only increase the self-efficacy of healthcare providers but also their confidence and decision-making abilities, enabling them to make quicker, more informed decisions that could be crucial for effective diabetes management. However, there is still a lack of information in this regard.

Considering the above comments, a holistic and integrated advanced training program on type 2 diabetes, based on the most recent clinical guidelines and the latest advancements in diabetes management, has been developed to improve the knowledge and self-efficacy of primary care professionals. The aim is to increase the performance of diabetes self-management support. In addition, we aim to evaluate the effectiveness of this educational program on the metabolic parameters of patients with type 2 diabetes, in order to confirm the improvement in patient care.

## 2. Materials and Methods

### 2.1. Design

A longitudinal, quasi-experimental, analytical, and comparative study with a control group was carried out in primary care settings. A training program for nurses and physicians was developed and performed, including clinical guidelines and novel approaches to type 2 diabetes (Supplementary Information S1). The program was focused on diabetes therapy and its management for individual patients. We focused on updating knowledge on various topics, including new drug therapies and engaging health professionals in case study simulations. These components aim to provide healthcare professionals with the necessary skills to provide evidence-based practice and patient-centered care. The whole study was carried out from January 2022 to June 2023.

The course featured eight three-hour sessions and was overseen by a Diabetes Nurse Practitioner (DiNP). Topics covered by the course included a review of the current treatment algorithms, oral therapy and insulin injections, diabetes complications (including cardiovascular risk factors and treatment of diabetic foot), glucose monitoring, responsiveness and motivation, diabetes in pregnancy, and the role of the multi-professional team. Attendance was mandatory for all sessions. In the second phase, patients were allocated depending on whether professionals followed the educational intervention or not. Therefore, a cohort study was conducted. The study adhered to the STROBE norms for cohort studies.

### 2.2. Primary Care Health Professionals and Educational Intervention

The study included 157 health professionals working in primary healthcare clinics (HMO Kupat Holim Meuhedet North Dist (Israel)), 77 PCPs (general practitioners and family physicians), and 80 nurses. The mean experience of the professionals was 15 ± 11 years (median = 13.5 years, IQR = 18). Professionals were divided into two groups depending on baseline knowledge about diabetes. The intervention group was composed of 77 health professionals (40 nurses, 37 practitioners), who participated in a diabetes education program. An additional group of 80 health professionals who did not participate in the education program (40 practitioners, 40 nurses) was considered as a control group. The selection criterion was to have at least 50 patients with type 2 diabetes under their care. As exclusion criteria, professionals with a previous specialty in diabetes and those who had previously taken similar courses on type 2 diabetes were excluded from the study.

The minimum required sample size was 48 individuals in each group, calculated based on a 95% confidence level and 10% confidence interval (minimum *n* = 96), according to the GPower 3.1 calculations [10].

The learning methodology of the educational program included theoretical exposition, flipped classroom, presentation and discussion of clinical cases, and problem-based learning through workshops. The training program comprised 8 mandatory sessions (with the option to attend a missed session in another of the 4 educational program groups that were implemented). The objective of the educational program was to engage a cohort of health professionals, comprising both doctors and nurses, in a collective learning experience. This was deemed essential to foster a collaborative approach, which is pivotal for effective chronic patient management within a healthcare context. Consequently, the program integrated a joint follow-up and roundtable discussion at its conclusion. The rationale behind the training program itself stipulates the necessity for joint training activities, rather than individual ones. This is due to the fact that the long-term follow-up of the patient represents a common objective shared by both professional profiles.

### 2.3. Data Collection and Instruments and Variables

Diabetes knowledge was measured with seven multiple-answer questions about a health professional knowledge about diabetes and its care, e.g., “The following answers regarding treatment with GLP1 drug group are correct”, and several clinical cases. Questions were developed based on current ADA guidelines and previous similar works [11,12]. The number of correct answers was considered as the previous diabetes knowledge score (range 0–7 points, where 0 indicates a complete absence of knowledge, and a score of 7 indicates the highest level of knowledge). The questionnaire yielded a Cronbach’s α of 0.63, which, although it would be desirable for it to be greater than 0.7, indicates acceptable internal consistency and is similar to previous studies [12].

Those who demonstrated lower levels of previous diabetes knowledge, considered as a score lower than 3.5 points, were included in the intervention group, while those who demonstrated higher knowledge were placed in the control group.

The self-efficacy test employed in the present study comprised six subscales (Supplementary Information S2): mastering knowledge of diabetes treatment, belief in treatment ability, knowledge required to begin treatment with SGLT2, knowledge required to begin treatment with GLP1, perceptions about patients with type 2 diabetes management, and the care delivered at the primary care center. In the mastering knowledge of diabetes (12 items) dimension, participants rated their proficiency in a range of competencies, including the ability to identify, diagnose, and manage diabetes. The belief in treatment ability (5 items) scale was used to evaluate participants’ confidence in their ability to identify conditions affecting diabetes balance, change drug treatments, and prevent acute conditions. Knowledge of the factors required to begin treatment with SGLT2 (7 items) and GLP1 (9 items) was based on decision making in clinical settings about the use of these drugs. The perceptions about type 2 diabetes management (12 items) referred to the opinions of professionals about how patient care should be, for example, whether it is mandatory to visit a nutritionist or whether nursing follow-up is mandatory. Finally, the care at the primary care center (7 items) evaluated the perception of the management of patients with type 2 diabetes at the workplace, e.g., “The level of treatment for diabetics in my clinic is insufficient”.

Participants rated the questions on a Likert scale from 1 to 5. Ratings were provided from 1 (not at all) to 5 (to a very large extent). A mean score was calculated for each dimension, defined as the mean value of all the items that made up that dimension, and an overall self-efficacy score was calculated as the mean value of all the items in the scale (52 items). All scores had a range of 1–5 points. Higher scores indicate greater self-efficacy in diabetes management and treatment, with scores of 5 reflecting enhanced self-efficacy. The Cronbach’s α of the questionnaire was 0.91, which indicates a very high internal consistency.

The test employed in the present work was developed ad hoc, mainly because several items of previous works about knowledge were old-fashioned and outdated for current type 2 diabetes management. However, the following scales were used as a starting point: the Diabetes Knowledge Questionnaire [13], the Revised Diabetes Knowledge Scale [14], the Diabetes Management Self-Efficacy Scale [15], and the Diabetes Empowerment Scale [16].

The last determination of health professionals was the perception of the quality of care. This scale evaluates the effectiveness and quality of diabetes care provided by healthcare professionals in different settings. Eccles et al. provided a framework (iQuaD) for assessing and enhancing the quality of diabetes care in various healthcare environments [17]. The present study employed an 18-item scale, with a Likert score ranging from 1 (disagree) to 5 (totally agree), based on two dimensions of the Eccles et al. iQuaD test: perceptions of treating diabetes in the community, which emphasizes understanding practice and staff characteristics that influence diabetes care in community settings, and the iQuaD dimension of treating patients with diabetes at the primary care clinic, which focuses on identifying factors that impact diabetes management quality at the primary care level. An overall quality of care score was determined as the mean score of all the items (range 1–5 points). The Cronbach’s α of the test was 0.87, also indicating a high reliability.

### 2.4. Patients with Type 2 Diabetes

Patients with a diagnosis of type 2 diabetes mellitus, HbA1c ≥ 7%, and an age range between 18 and 85 years, treated by physicians and/or nurses at several primary care settings took part in this longitudinal cohort study. Patients with a diagnosis of type 1 diabetes mellitus, those who were pregnant, those with severe disabilities, or those with high fluctuations in glycemic control (i.e., having more than 3 episodes of hyperglycemia and/or hypoglycemia in the 3 months before the beginning of the study), were excluded. Being treated by an endocrinologist or attending a diabetes clinic was also considered an exclusion criterion.

Patients were allocated to the intervention group and control groups depending on whether the primary care professional attended the advanced education program or not. Finally, 4099 patients took part in this phase of the study (2088 monitored by professionals included in the education group and 2011 monitored by professionals included in the control group).

### 2.5. Clinical Parameters

In order to evaluate the effectiveness of the education program on clinical outcomes of patients with type 2 diabetes, demographic and clinical data were collected from clinical records of patients with diabetes who were associated with each of the health professionals participating in the study.

These data were collected at baseline and 12 months after the end of the education program. Clinical parameters included different variables usually evaluated in primary care settings, including HbA1c levels, lipid metabolism parameters (total cholesterol, fasting triglycerides, non-high-density lipoprotein cholesterol), blood pressure, frequency of hypoglycemia incidents, frequency of hyperglycemia incidents, the number of purchases of prescribed diabetes medications, frequency of visits for laboratory testing, the number of appointments at the clinic attended by the patient, the number of appointments with dietitian attended by the patient, and the frequency of foot check-ups. Other variables included age, sex, year of diagnosis, and the presence of co-morbidities.

### 2.6. Ethics

All patient data were anonymized and coded to prevent identification. The study adhered to the ethical principles outlined in the Declaration of Helsinki, emphasizing respect for individuals, beneficence, and justice. Ethical approval was obtained from the local health service (code: 529/2021, date: 4 November 2020), which included a detailed study description, including design, procedures, risks, benefits, cultural and social implications, and data handling. The necessary permissions were requested before gaining access to patients’ clinical data.

Regarding primary care staff, permission was also requested for the implementation of the training program, which required the application of different questionnaires adapted to the local language.

### 2.7. Statistical Analysis

Data were represented as mean ± sd unless otherwise indicated for quantitative variables and frequency (%) for qualitative variables. Once normality was confirmed through the Kolmogorov–Smirnov test, *t*-tests were conducted to evaluate possible differences at baseline in age and other parameters. Changes from the baseline were evaluated by paired *t*-tests. Moreover, to compare the changes between those patients managed by health professionals who followed the advanced practice education versus those who did not, an independent sample *t*-test was conducted comparing mean changes (6-month values minus baseline values).

Additionally, an ANCOVA analysis was conducted to adjust the significance level for several covariates such as sex, age, and other baseline parameters. To confirm the effect on the main clinical variables (fasting glucose and HbA1c) while considering that patients were grouped by primary care specialist, who were divided into intervention and control groups, a mixed-effect model was conducted to capture variability within primary care staff and between groups of patients. Primary care staff was considered as a random effect, while the educational intervention was considered as a fixed effect. To do this, change in metabolic parameters—fasting glucose and HbA1c—was established as the dependent variable, and the intervention group was the fixed independent variable. The primary care staff was treated as the random variable. The mathematical model used was as follows:

(1) $$y_{ij}=\beta_{0}+\beta_{1}\cdot Group+u_{j}+\in_{ij}$$

where *y_ij_* means the change in the metabolic parameter, *β*_0_ is the mean change in the parameter without intervention, *β*_1_ is the effect of educational intervention, *u_j_* is the random effect for primary care staff *j*, and ∈*_ij_* is the residual error for patient *i* of primary care staff *j*.

To verify that the assumptions of normality were met in the adjusted model, a study of the residuals was carried out. Additionally, a comparison analysis was performed with a null model (not including intervention groups), using a likelihood ratio test to verify whether the inclusion of the group variable significantly improved the model.

Finally, since primary care professionals were not randomly assigned to the educational intervention group, a propensity score matching (PSM) analysis was used to reduce selection bias and balance the characteristics of patients cared for by health professionals who received the educational intervention and those cared for by professionals who did not (control group). To adjust for baseline differences between groups, the PSM technique was applied using the MatchIt package in R. The propensity model was built using logistic regression where the dependent variable was primary care participation in the educational intervention and the independent variables were the baseline characteristics of physicians and patients (including age, previous experience, baseline knowledge about diabetes, socioeconomic status, and patient clinical variables such as blood glucose and HbA1c).

The nearest neighbor matching method was employed with a 1:1 ratio and without replacement. The balance between groups before and after matching was assessed using propensity score distribution plots and a summary of covariate balance. After matching, baseline differences between groups were significantly reduced, ensuring adequate comparability for analysis of intervention effects.

Once the matched sample was obtained, a comparative analysis of clinical outcomes in patients was performed, primarily assessing reduction in glycated hemoglobin (HbA1c) levels. To do so, a *t*-test was used to compare the means of the HbA1c change variable between patient groups treated by health professionals who participated in the educational intervention and those who did not.

The significance level was established at *p* < 0.050. All analyses were carried out with IBM SPSS 28.0 software and R software (4.4.2 release) (see Supporting Information S4 for R code).

## 3. Results

### 3.1. Previous Knowledge

**Figure 1:**
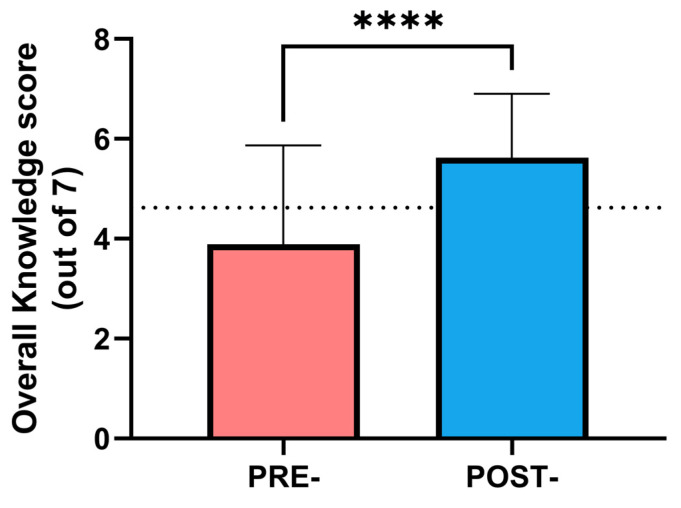
Changes in Knowledge about Diabetes score (out of 7) after the advanced education program in the professionals allocated in the intervention group. The dotted line represents the mean score of the control group. Statistical differences were evaluated through a paired *t*-test. **** *p* < 0.0001.

One hundred and sixty primary care health professionals were evaluated to analyze previous knowledge about diabetes through a 7-item survey. The mean score obtained by these professionals was 4 (IQR:3), which indicates that the professionals had an average knowledge of basic diabetes concepts (Figure 1). As per protocol, professionals were divided into control (those with higher knowledge) and intervention (those with lower knowledge) groups, depending on whether they received diabetes education or not. After the educational intervention, knowledge of diabetes management was significantly improved, as the mean score of the survey changed from 3.2 to 5.6 points in these professionals (*p* < 0.001). This improvement was independent of sex, age, and professional experience (*p_adj_* = 0.002) (Figure 1).

### 3.2. Self-Efficacy

The overall self-efficacy of the participants was high, with a mean score of 3.7 ± 0.4 (out of 5 points). Questions related to drug treatment showed a high mastery level, although the highest scores were observed in staff perception regarding diabetes treatment in the community. For instance, 67.5% of the participants agreed that a new diagnosis was a window of opportunity to balance diabetes, and 54% agreed that every patient must be referred to a nurse. In contrast, only 2% of the participants considered that the level of treatment for diabetes in the clinic was insufficient.

**Figure 2:**
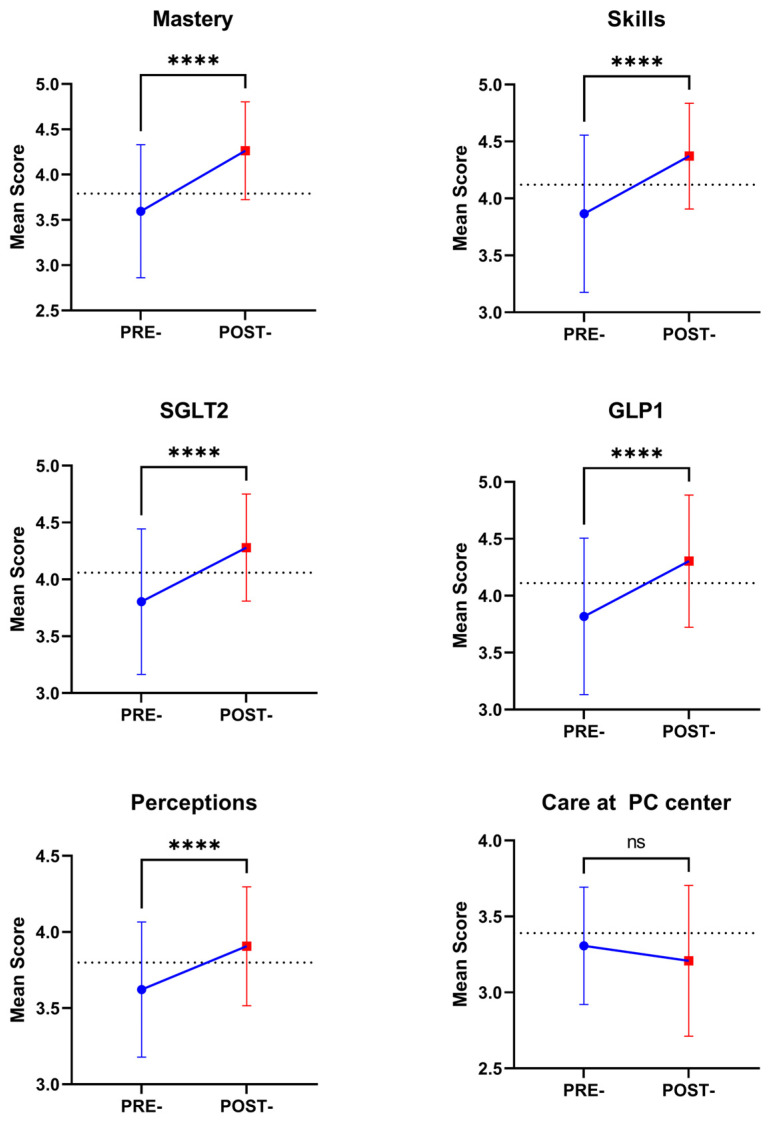
Changes in the mean score of the different dimensions of the self-efficacy test (out of 5) after the advanced education program. The dotted line represents the mean score of the control group. Statistical differences were evaluated with a paired *t*-test. **** *p* < 0.0001; ns: not significant differences.

When specific dimensions were evaluated (Figure 2), the highest score was obtained in the Skills dimension (3.9 ± 0.7), while the lowest score was observed in the Care at the primary care clinic dimension (3.3 ± 0.4). Baseline values of all dimensions were higher in the control group (*p* < 0.001 in all cases). However, our data showed an improvement in the global score, as well as in all dimensions (*p* < 0.001 in all cases), except in the Care at the primary care clinic dimension (*p* = 0.761), where there were no differences with baseline values of the control group and at the end of the intervention (Figure 2).

### 3.3. Self-Perceived Quality of Care

**Figure 3:**
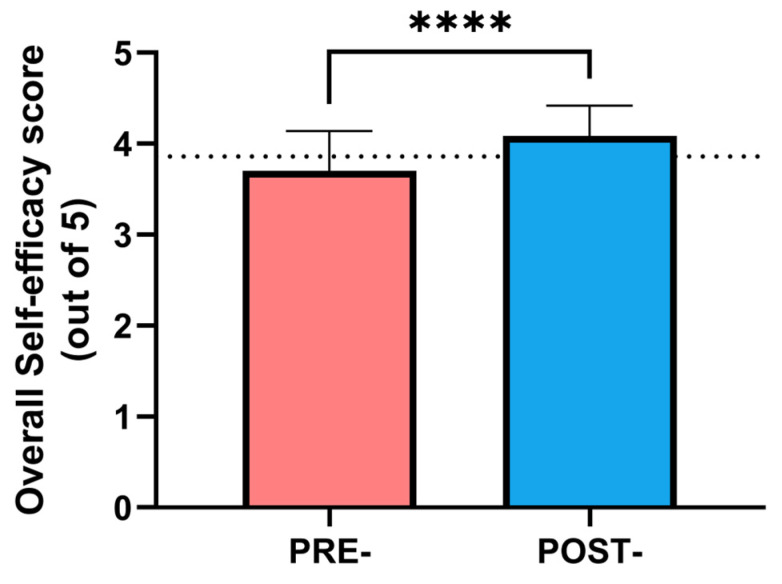
Changes in perceived quality of care score (out of 5) after the advanced education program in the professionals allocated in the intervention group. The dotted line represents the mean score of the control group. Statistical differences were evaluated through a paired *t*-test. **** *p* < 0.0001.

From the self-perceived quality of care test data (Figure 3), the baseline scores in both the control and education intervention groups were similar (4.1 ± 0.4 and 3.8 ± 0.4). Again, the educational intervention significantly improved the score obtained in the test, changing from 3.8 to 4.2, which represents a mean improvement of 10% (*p* < 0.05).

### 3.4. Baseline Clinical Characteristics of Patients with Type 2 Diabetes

Finally, 4099 patients took part in the study, of which 2011 were allocated to the control group and 2088 to the intervention group. The mean age was 64 years, and the sex proportion was similar (48% men and 52% women). The data shown in Table 1 present the age and other baseline clinical characteristics of the patients. Age was similar in both groups, as well as all the parameters related to lipid metabolism. Although weight was similar, BMI was slightly higher in the control group, as well as the duration of diabetes (7 years vs. 6 years) and the diastolic blood pressure. In contrast, the filtration rate (eGFR) was higher in the intervention group. Moreover, fasting glucose and HbA1c values were also similar. It is important to note that although the raw differences were minimal, the large population size tends to describe a statistically significant difference, as occurred with the HbA1c, where the difference observed (0.2% between groups) had low clinical significance. Nevertheless, these features were included in the ANCOVA analysis, as described later.

**Figure 4:**
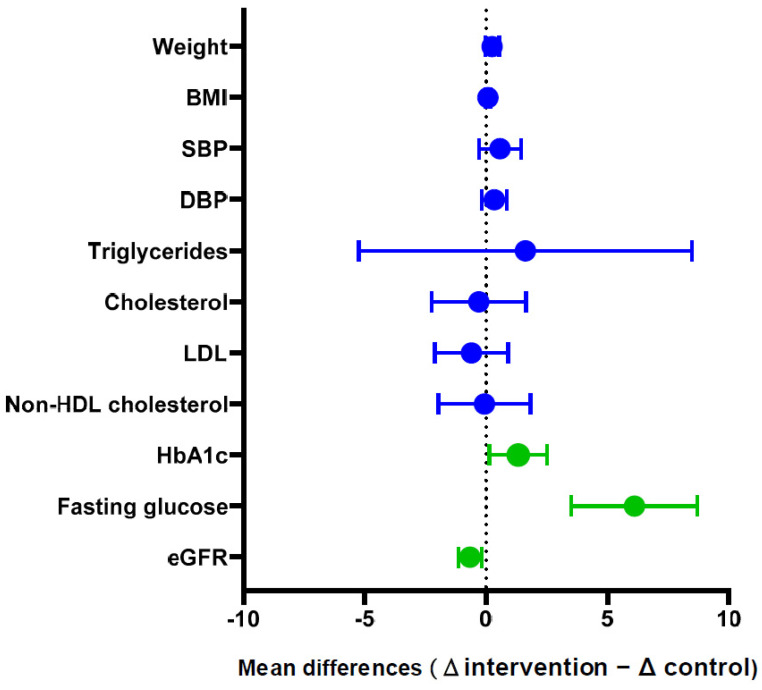
Forest plot showing changes in metabolic parameters (mean differences) between those patients treated by clinical staff of intervention group and by clinical staff in the control group. Each point represents the mean difference and the error bars indicate the 95% confidence interval. A positive value indicates greater improvement in the intervention group compared to the control group. Those variables with statistically significant differences are shown in green.

To test the hypothesis of better metabolic control in the patients managed by professionals who underwent advanced practice education (experimental group), the clinical parameters were evaluated after the intervention. Detailed mean changes are described in Supplementary Table S1. After 12 months, both control and experimental groups experienced an improvement in most metabolic parameters, except for total cholesterol and LDL plasma levels, which remained unaltered after the intervention. To compare the effectiveness of diabetes management, mean changes were compared through a forest Plot (Figure 4). The main improvement as a consequence of the intervention was observed in fasting glucose, although the improvement in glycosylated hemoglobin and renal function was also significantly higher in the experimental group.

A linear mixed model (MLM) was performed to assess the effect of training program participation on HbA1c and fasting glucose. Primary care professionals were considered as random effects, while educational training was treated as a fixed effect. For HbA1c, the restricted information criterion of maximum likelihood (REML) converged at 10,594.4, indicating model fit. Regarding the random effects, an intercept variance of 0.003666 was observed for the health professionals’ random effect, with a standard deviation of 0.06055, indicating that variability among physicians is low. Regarding the fixed effects, participation in the educational training program was found to have a significant effect on HbA1c (estimate: −0.19793, s.e.: 0.03131, t = −6.321, *p* < 0.001), suggesting that patients cared for those professionals in the intervention group achieved better clinical outcomes, reflected in a reduction in the HbA1c.

In summary, the results suggest that participation in the training program had a positive and significant impact on clinical outcomes, adjusting for variability between physicians. To confirm the adequacy of the MLM, a likelihood ratio test was performed. The null model included only the intercept with a random effect for the health professionals (null_model2), while the full model included the participation variable as a fixed effect (model2), in addition to the random effect for the professionals. The full model showed a significantly better fit compared to the null model (χ^2^ = 39.746, *p* < 0.001). The AIC (10,591 for the full model vs. 10,629 for the null model) and BIC (10,616 for the full model vs. 10,647 for the null model) also favored the full model, indicating that the inclusion of the participation variable substantially improved the fit of the model to the data. The results were similar for fasting glucose levels (Table 2).

These results reinforce the importance of training participation as a significant predictor of changes in the clinical parameters evaluated (HbA1c and fasting glucose levels), since its inclusion in the model resulted in a significant improvement in the fit according to the information criteria and the likelihood ratio test.

**Figure 5:**
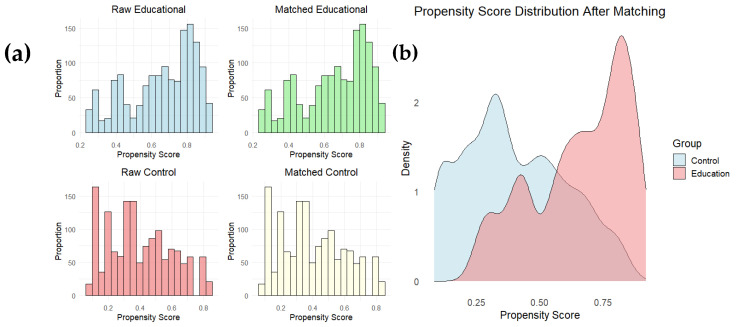
(**a**) shows the histograms of the propensity score distribution for the educational intervention and control groups, before and after matching. The figure shows how the initial differences between the two groups are reduced after propensity score matching. (**b**) shows the density plot of the propensity score after matching, showing the overlap between the educational intervention and control groups. The greater overlap indicates a good balance between the groups, which improves comparability between them.

Finally, Figure 5a,b shows the distribution of propensity scores before and after matching for both groups (educational intervention and control). Figure 5a shows histograms comparing the distributions of the propensity scores in the groups before and after matching, showing that the initial differences between the groups decreased significantly after matching. Figure 5b shows the superposition of the propensity score densities between the treated and control groups after matching, indicating an adequate balance between both groups after applying the PSM.

The post-matching analysis showed significant differences in clinical outcomes between patients treated by physicians who participated in the educational intervention and those who did not participate. In particular, a greater reduction in HbA1c levels was observed in patients in the intervention group (mean: −0.33) compared to patients in the control group (mean: −0.11), with a mean difference of 0.21 (95% CI: 0.14 to 0.29, *p* < 0.001). Welch’s *t*-test confirmed that this difference was statistically significant (t = 5.452, *p* = 5.418 × 10^−8^), suggesting a positive impact of the educational intervention on patients’ clinical outcomes.

## 4. Discussion

The present work was conducted to evaluate the effectiveness of an integrated advanced training program on type 2 diabetes for primary care professionals, oriented to improve their skills in managing patients with type 2 diabetes. The first step to confirm the effectiveness of any educational intervention is the evaluation of the acquired information. Surprisingly, we observed significant differences among professionals regarding baseline knowledge about type 2 diabetes management. This finding led us to target the education intervention to those with insufficient knowledge.

Several previous works have evaluated the knowledge of health professionals in primary care settings, yielding diverse results. Torres et al. revealed that primary healthcare professionals in Brazil lack preparation and technical knowledge for effective patient education on type 2 diabetes [18]. However, this study was conducted several years ago, and diabetes care has significantly changed since then. Another study focused on endocrine physicians showed suboptimal knowledge and practices regarding diabetes management, underscoring the need for education initiatives and guideline dissemination [19].

More recent studies, such as the work by Mohammed et al., revealed more promising data. About 66.7% of primary care physicians demonstrated good knowledge of routine diabetes care [20]. Increasing knowledge about the disease is essential to provide adequate care. This is particularly critical in primary care, where long-term care is provided. Knowledge gaps in this setting can persist over time if not addressed.

A systematic review emphasized the importance of addressing knowledge gaps, clinical inertia, and patient adherence to improve diabetes care in primary care [21]. As type 2 diabetes is a complex chronic condition, providing patients with the knowledge and skills to manage their clinical condition helps them achieve correct metabolic control over time. This approach also prevents complications and improves patient quality of life [22]. Thus, increasing staff knowledge is a prerequisite for adequate patient management.

Diabetes is one of the leading causes of death, and therefore, a healthcare priority. Collaborative, interdisciplinary care can provide better management for chronically ill patients, especially in primary care settings [23]. The global rise in diabetes prevalence, along with the complications associated with poor glycemic control, necessitates more effective management strategies. These strategies should include enhancing the self-efficacy of primary care professionals to empower patients with type 2 diabetes. By fostering self-efficacy, professionals can help patients better manage their condition, improving outcomes and reducing healthcare interventions.

It is important to differentiate between self-management and self-efficacy. The former refers to the care of the patient. Self-efficacy, first defined by Bandura in 1977, refers to an individual’s confidence in their ability to perform tasks and solve problems in their work context [24]. A successful diabetes care team should demonstrate high self-efficacy, which is linked to improved patient outcomes and greater professional satisfaction [25]. Higher self-efficacy among health professionals is associated with better patient adherence to diet, physical activity, and blood glucose monitoring [26].

Factors such as educational level, social support, and clear communication from healthcare providers are associated with improved health literacy and self-efficacy [27]. Interventions based on self-efficacy theory can enhance health literacy, self-efficacy, and self-care behaviors in patients with diabetes [28]. By promoting patients’ self-efficacy, health professionals can empower patients to take a more active role in their treatment, leading to better disease control and prevention of serious complications [29].

In this study, health professionals showed high baseline self-efficacy, especially regarding their perception of community diabetes treatment. Since the reform of health systems carried out after the WHO conference in Alma Ata (1978), the strategy of primary care began to implement health programs to address chronic conditions (such as diabetes type 2), which should be developed by the primary care team, being physicians and nurses the basic core for medium- and long-term patient control [30]. This is an important issue since previous works have demonstrated that self-efficacy is a strong predictor of diabetes self-management [31]. Most importantly, self-efficacy was significantly improved after the education intervention, so an increase in the quality of life of the patients would be expected.

Previous studies have demonstrated the effectiveness of educational interventions in improving self-efficacy among patients with type 2 diabetes [32,33]. However, evidence of the impact of training for health professionals and its effect on the metabolic control of their patients is still emerging. Continuous education for healthcare professionals is crucial, as evidence shows that training programs improve the metabolic control of patients with type 2 diabetes. Studies indicate that structured educational interventions for healthcare providers significantly enhance clinical outcomes, including better glycemic levels, adherence to treatment guidelines, and patient self-management skills.

Incorporating continuous education into professional development ensures the implementation of evidence-based practices in clinical settings. This approach offers long-term benefits in patient care. Regular training equips healthcare providers to address the complexities of diabetes care and fosters a proactive approach to preventing complications and reducing the overall burden [34,35].

Initially, self-efficacy among healthcare staff showed averaged scores across all dimensions. However, the educational program increased the mean score of all measurements, indicating improved self-efficacy in managing patients with type 2 diabetes. One exception was the dimension related to the management of patients at the primary care center, which showed no improvement. This dimension includes essential questions, such as whether medication is adjusted to glucose level or if long-term insulin should be given only at night. These critical aspects of care are mandatory for any professional who cares for these patients.

Although the education program successfully improved self-efficacy, specific areas still require further attention. Most prior studies evaluating educational interventions to improve self-efficacy in type 2 diabetes focus on patients rather than healthcare professionals [36,37,38]. Consequently, there was limited certainty about whether improving professionals’ self-efficacy would also enhance patient care quality.

When assessing improvements in the quality of care, this can be approached from two perspectives. This study is pioneering because it simultaneously assessed health professionals before and after the intervention and followed up with their patients. Previous research in this area often evaluates patients and professionals separately [39]. To our knowledge, previous studies on this topic are limited to review papers that evaluate these aspects in patients and professionals separately [40,41], which reinforces the suitability of the present work.

From the professionals’ perspective, the perception of care quality—defined not only as having disease knowledge but also ensuring its proper delivery to patients—was significantly improved. This improvement was observed in the scores of perceived quality of care among health professionals who participated in the education program. Regarding patients, key parameters in type 2 diabetes control, such as fasting glucose, glycosylated hemoglobin, and glomerular filtration rate (eGFR), showed significantly greater improvements in those managed by trained professionals compared to professionals from the control group.

Overall, these findings indicate better type 2 diabetes management in patients treated by professionals who participated in the education program. Propensity score matching minimized baseline differences between comparison groups, ensuring that clinical outcome improvements were not due to baseline differences in physician or patient characteristics. The results suggest that the educational intervention, which targeted professionals with low baseline knowledge of diabetes management, positively impacted HbA1c levels in their patients. This highlights the importance of ongoing education for improving clinical care quality. Diabetes education is essential for enhancing health outcomes in type 2 diabetes. To achieve this, patients must feel confident in managing their disease [42]. These results confirm the adequacy of the education program.

As in any research, the present work has limitations that deserve consideration. The use of a non-validated test for certain variables may limit the generalizability of these results. This issue arises because no validated versions exist in Hebrew, the original language of the study. Furthermore, type 2 diabetes is a condition with a constantly evolving therapeutic approach. This required introducing specific items related to the management of newer drugs, such as SGLT2 inhibitors and GLP1 analogs, which are absent in previously validated tests.

Additionally, no specific tools for primary care fit the objectives of the study. However, the high-reliability values from our data suggest the results obtained are reliable. A potential bias could arise from selecting providers with lower baseline knowledge for the intervention. This could be seen as a selective bias, but it was necessary to target those most in need of education.

A higher baseline HbA1c level in the patients from the intervention group could also be a potential bias, as it indicates more room for improvement. However, the ANCOVA analysis, which adjusted for this confounder, did not alter the significance of the effect of the intervention.

Finally, the training sessions were conducted outside working hours, lasting three hours per week. This scheduling may represent a limitation, as it may have negatively impacted participation in the program. Regarding sample size, although the patient cohorts were adequate, the number of professionals was smaller. Nevertheless, it was sufficient to meet the sample size requirements of the study.

## 5. Conclusions

The present advanced education program oriented to primary care health professionals has increased knowledge, self-efficacy, and perceived quality of care, which was mirrored in the better metabolic control of patients with type 2 diabetes, as mirrored in an improvement in fasting glucose, glycated hemoglobin, and glomerular filtration rate, some of the keystones of diabetes control. Interestingly, the improved care provided by professionals in the intervention group—those with initially lower knowledge—emphasizes the importance of regular evaluation and training, particularly for high-demand conditions like type 2 diabetes. However, certain aspects, such as routine care in primary care consultations and patients’ lipid metabolism, showed no significant improvement. Despite this, the study serves as a foundation for educational initiatives aimed at enhancing the quality of care provided by primary care professionals to patients with type 2 diabetes. Notably, this is one of the few studies demonstrating that improving the knowledge and self-efficacy of primary care professionals directly benefits patients with type 2 diabetes.

## Figures and Tables

**Table 1:** Baseline clinical and sociodemographic characteristics of patients with type 2 diabetes according to the allocation group of their health professional in charge.

Characteristics	Total Population (n = 4099)	Control Group (n = 2011)	Intervention Group (n = 2088)	*t*	*p* (Sig)
Age (years)	63 ± 11	64 ± 11	63 ± 12	0.798	0.424
Weight (kg)	85.3 ± 16.5	85.8 ± 16.6	84.8 ± 16.4	1.828	0.068
BMI (kg/m^2^)	30.11 ± 6.88	30.57 ± 6.94	29.67 ± 6.80	3.991	<0.001
Duration of diabetes (years)	6 ± 2	7 ± 2	6 ± 2	2.047	0.041
SBP (mmHg)	131 ± 16	131 ± 15	131 ± 16	−0.199	0.842
DBP(mmHg)	76 ± 9	76 ± 8	75 ± 9	2.373	0.018
Fasting glucose (mg/dL)	135 ± 47	135 ± 45	136 ± 50	−0.748	0.454
HbA1c (%)	7.1 ± 3.1	7.0 ± 1.4	7.2 ± 4.1	−2.533	0.110
Triglycerides (mg/dL)	166 ± 140	166 ± 166	167 ± 110	−0.313	0.754
Total cholesterol (mg/dL)	173 ± 42	173 ± 44	173 ± 41	−0.070	0.944
Non-HDL (mg/dL)	129 ± 41	129 ± 42	129 ± 40	−0.647	0.518
LDL (mg/dL)	97 ± 34	97 ± 35	97 ± 34	0.048	0.962
eGFR (mL/min/1.73 m^2^)	79.7 ± 19.7	78.8 ± 19.6	80.6 ± 19.8	−2.787	0.005

Data represent mean ± sd. BMI: body mass index; SBP: systolic blood pressure; DBP: diastolic blood pressure; HDL: high-density lipoprotein; LDL: low-density lipoprotein. eGFR: estimated glomerular filtration rate. Differences between control and intervention groups were evaluated using a *t*-test.

**Table 2:** Data derived from the mixed effect analysis regarding HbA1c and fasting glucose.

Dependent variable HbA1c (fixed effects):							
	Estimate		Std. Error	df		t value	Pr (>|t|)
Intercept	−0.08832		0.02295	578.71981		−3.849	0.000132
Group	−0.19793		0.03131	3680.69538		−6.321	2.91 × 10^−10^
Likelihood ratio test:							
	Npar	AIC	BIC	logLik	Chisq	Df	Pr (>Chisq)
Null_model	3	10,629	10,647	−5311.3			
Model	4	10,591	10,616	−5291.5	39.746	1	2.893 × 10^−10^
Dependent variable fasting glucose (fixed effects):							
	Estimate		Std. Error	df		t value	Pr (>|t|)
Intercept	−3.312		0.984	553.687		−3.365	0.00818
Group	−6.094		1.321	3737.442		−4.614	4.07 × 10^−6^
Likelihood ratio test:							
	Npar	AIC	BIC	logLik	Chisq	Df	Pr (>Chisq)
Null_model	3	18,988	19,007	−9491.2			
Model	4	18,979	19,004	−9485.5	11.461	1	0.0007108

## Data Availability

Data are available at Mendeley data with the following reference: Hernandez Morante, Juan Jose (2024), “Type 2 diabetes—Educational program in Primary Care”, Mendeley Data, V1, doi: 10.17632/k7fs8hh7vn.1.

## Supplementary Materials

The following supporting information can be downloaded at https://www.mdpi.com/article/10.3390/nursrep14040280/s1, Supporting Information S1: educational program Information, Supporting Information S2: questionnaire employed to collect data from primary care professionals, Supporting Information S3, Supplementary Table S1: clinical parameter changes after intervention in both the control and experimental groups, Supporting Information S4: R code.

## Public Involvement Statement

No public involvement in any aspect of this research.

## Guidelines and Standards Statement

This manuscript was drafted against the STROBE (Strengthening the Reporting of Observational Studies in Epidemiology) for cohort-study research.
