# Supplementary material for: Increasing Self-Efficacy for the Management of Patients with Type 2 Diabetes Through an Advanced Practice Education Program for Primary Care Professionals
Source: Nurs Rep. 2024 Dec 5;14(4):3830–46. doi: 10.3390/nursrep14040280 (PMC11676394; doi:10.3390/nursrep14040280)
Supplement: Supplementary file 1 [file nursrep-14-00280-s001.zip › nursrep-3282724-supplementary.pdf]

INCREASING SELF-EFFICACY FOR THE MANAGEMENT OF PATIENTS WITH TYPE 2 DIABETES THROUGH AN ADVANCED PRACTICE EDUCATION PROGRAM FOR PRIMARY CARE PROFESSIONALS

SUPPLEMENTARY MATERIALS

Supporting Information S1. Educational program Information

The objective of the educational programme was to enhance the knowledge baseline primary care professionals with regard to the optimal management of diabetes, as part of a future initiative to improve the quality of diabetes care at the provincial level.

The training programme comprises a series of sessions. 8. It was mandatory for all participants to attend all sessions.

It was recommended that cases for consultation be presented by the attending health professionals.

It was recommended that health professionals apply the instructions and lessons learned during each session, commencing from the outset. Complex cases were presented for discussion as part of the training programme.

The venue and date of the event are as follows: The sessions were held on Mondays between 6:30 pm and 9:30 pm. Each session lasted three hours and was developed at the county executive office. The meetings were conducted by a provincial diabetes forum, which included the participation of diabetes consultants. After the conclusion of the course, a follow-up and roundtable discussion were conducted.

The Diabetes School Program

The first session of the programme commenced with the following agenda items: "Setting a Diabetes Framework". Greetings and opening remarks – District Medical Director, District Manager. A review of the updated therapeutic algorithm in type 2 diabetes of 2021, with an emphasis on setting treatment goals and preventing cardiovascular morbidity. A review of oral medication, with guidance on selecting the most appropriate drug for the patient.

Second session. "The right shot for the right patient"

Basal Insulin / GLP-1: instructions for selecting the most appropriate injectable drugs for each patient

Third Session: "Diabetes Complications - Recent Updates" Cardiovascular complications - CVOT studies Diabetic kidney and the effect of diabetes drug treatments on kidney disease - how to protect the kidney beyond ACE and ARB The nurse's role in a primary care clinic for optimal care and follow-up

Fourth Session: "Diabetes Complications - Diabetes Is Not Just Sugar - Cardiovascular Risk Factors" Dyslipidemia - important progress in the treatment besides statins Balancing blood pressure in diabetics - new guidelines Case discussions

Fifth Session: "Technology, Motivational Interview, Obesity and Pre-Diabetes" Technology tasting and diabetes - Continuous monitoring of blood glucose levels- Beyond glycosylated hemoglobin. Tools for improving the responsiveness of medical clinics - a motivational interview Obesity and pre-diabetes - Can diabetes be prevented, is there room for pre-diabetes medication

Sixth Session: "Diabetic Foot" Diabetic neuropathy - diagnosis, treatment Diabetic foot - the nurse's role, manner of examination and follow-up according to current guidelines

Seventh Session: "Pregnancy and Diabetes" Pregnant hyperglycemia - pre-pregnancy diabetes / gestational diabetes - diagnosis and treatment Nurse monitoring of gestational diabetes in primary care

Eighth Session: "Multi-Professional Team" Dieticians and their role in managing the treatment Social workers and their role in managing the care Physiotherapists and their role in managing the treatment Health workshops - their contribution in providing tools to deal with diabetes Certificate distribution and summary feedback

Supporting Information S2. Questionnaire employed to collect data from primary care professionals.

Part I. General Information

Questionnaire number ____________ (for office completion),
Date___ ____ ______

Staff Questionnaire: Treatment of diabetes in the community for the purpose of testing self-efficacy and knowledge in the field

Dear staff member, greetings!
You are asked to participate in a study aimed at examining the **Relationship between the contribution of diabetes training by a clinical nurse specialist in diabetes and the Diabetes Forum for satisfaction, self-efficiency and knowledge update in managing Diabetes treatment in Meuhedet health fund, and the subsequent quality of diabetes care in the Northern District.**

The questionnaire is anonymous. The information will be used for research purposes only. The questions and statements in the questionnaires are worded in masculine language but refer to both genders.

<u>Your opinion is important for future improvement in the diabetes school program</u>
Thanks for the cooperation!

Part A. Demographics:
Please, fill in the blanks or circle the correct answer:

Type of employment: 1) Self-employed 2) Employee
Sex : Male /Female
Age ____________
Professional experience in years__________
Did you get any Professional training in Diabetes YES / NO __________

Part II. Previous Knowledge

In this section, you are asked to answer the following 7 questions, choose the most appropriate answer to each question:

1. **The following answers regarding treatment with GLP-1 drug group are <u>correct EXCEPT for:-</u>**
   A. Inhibits glucagon secretion
   B. Accelerates insulin release
   C. Accelerates gastric emptying
   D. Reduces the feeling of hunger

2. **Side effects of treatment with ACTOS Includes: everything <u>EXCEPT</u>:**
   A. Weight gain
   B. Edema
   C. Can cause liver dysfunction
   D. Decrease in HDL
   E. Increases the chance of bone fractures.

3. **DPP4 Inhibitors vs. Agonists of GLP1RA:**
   A. Lowers HBA1C equally
   B. Lose weight equally

C. Causes far fewer side effects
D. Safer cardiovascular aspect

4. **58 years old, with a background of type 2 diabetes, receiving treatment with metformin and insulin according to the basal-bolus regimen. Released from the hospital a month ago with a recommendation to add EMPAGLIFLOZIN to the treatment after being hospitalized due to myocardial infarction. The patient turns to you due to general weakness and abdominal pain. He is panting – has deep and fast breaths. Sugar is 220 mg / dl. How will you act?**

A. I will calm him down. The patient appears to be having an anxiety attack
B. I will check for ketones in the urine, if negative, I will send him home
C. Probably dehydrated , I'll send him to the emergency room for a Saline IV
D. I'll send him to the emergency room, with suspicion of diabetic ketoacidosis.

5. **Dan, 35, is single. Diagnosed two years ago with type 2 diabetes. No background disease, no diabetes is known in the family . HbA1C = 8.5% (half a year ago HbA1C = 7.2%), BMI = 22kg / h^2. Treated w. Eucrease 50/500 X 2/d.According to him, he engages in physical activity and adheres to a healthy diet. Complains of signs of hyperglycemia. How would you define his condition and what is the recommended treatment?**

A. Worsening the balance of type 2 diabetes, it is recommended to add a type of GLP1 agonist treatment
B. As it seems it is probably a type of LADA diabetes therefore it is recommended to start insulin therapy and stop oral therapy.
C. The worsening of the balance of type 2 diabetes is apparently due to not adhering to a healthy lifestyle so additional oral medication should be added.
D. Apparently this is a type of LADA diabetes and therefore it is recommended to add a prep. from the SGLT2 family

6. **For a patient who complained of symptoms of hyperglycemia, a random blood test was performed in which a sugar value above 215 mg% was found. The result indicates:**
A. Carbohydrate Intolerance - IGT (Impaired Glucose Tolerance)
B. Perform a diabetes load test
C. Diagnosis of type 2 diabetes
D. Diagnosis of type 1 diabetes

7. **A patient who is overweight and has no background diseases, underwent a recent blood test and was found to have fasting blood sugar levels of 120 mg /% and glycated hemoglobin of 7.2%. The doctor gave a prescription for Metformin 850 mg 3 / day Tab. What are the problems with providing this treatment?**
A. The dose of the drug should be increased gradually (over about a month) due to fear of hypoglycemia.
B. The dose of the drug should be increased gradually (over a period of about a month) due to concern about gastrointestinal side effects.
C. The dose of the drug should be reduced to once a day due to fear of weight gain with the drug and the patient's weight should be monitored
D. Giving three servings a day may impair the patient's liver function.

Part III. Degree of Self-Efficacy

The following statements express areas of knowledge regarding diabetes. You are asked to indicate your degree of mastery of knowledge regarding the diagnosis and treatment of diabetes as reflected in your current position, according to the following scale:

Not at all	Slightly	Moderately	To a large extent	To a very large extent
1	2	3	4	5

	Not at all	Slightly	Moderatel y	To a Large Extent	To a very large extent
I master the knowledge about ...	1	2	3	4	5
Detection and diagnosis of diabetes	1	2	3	4	5
Identification, treatment and diagnosis of hypoglycemic state	1	2	3	4	5
Starting insulin treatment	1	2	3	4	5
Starting treatment with GLp-1	1	2	3	4	5
Decision on choosing oral therapy for diabetes	1	2	3	4	5
Decision on target value	1	2	3	4	5
HbA1C per patient	1	2	3	4	5
Decision on target values in fasting and two hours after food	1	2	3	4	5
Control of short - lasting and long lasting insulin types	1	2	3	4	5
Control of the GLP-1 types	1	2	3	4	5
Control of all oral therapies	1	2	3	4	5
Control of up-to-date guidelines for treatment selection including research update	1	2	3	4	5
Identification of complications and damage to target organs	1	2	3	4	5

The following statements relate to belief in your ability to perform ongoing tasks in the context of your role in the community according to the scale from 1 - Not at all up to 5 – To a very large extent.
I believe I can ....

	Not at all	Slightly	Moderately	To a Large Extent	To a very large extent
Identify a factor that affects the balance of diabetes	1	2	3	4	5
Identify situations that cause a state of imbalance to the patient	1	2	3	4	5
Medication change	1	2	3	4	5
Prevent hypoglycemic conditions in the patient	1	2	3	4	5
Effectively treat severe hypoglycemic condition	1	2	3	4	5

You are asked to address the following statements, which examine the knowledge required to begin treatment with SGLt2. To what extent will the following statements influence the decision to choose the treatment?

	Not at all	Slightly	Moderately	To a Large Extent	To a very large extent
A patient who is a bus driver	1	2	3	4	5
Patient with a cardiovascular background	1	2	3	4	5
Elderly patient	1	2	3	4	5
A Patient with additional background diseases (comorbidities)	1	2	3	4	5
HbA1C Level	1	2	3	4	5
High fasting sugar values	1	2	3	4	5
GFR between 45 and 60	1	2	3	4	5

You are asked to address the following statements, which examine the knowledge required to begin treatment with GLP-1. To what extent will the following statements influence the decision to choose treatment?

	Not at all	Slightly	Moderately	To a Large Extent	To a very large extent
Treated with BMI 28 and HbA1C = 7.5%	1	2	3	4	5
Patient with a cardiovascular background	1	2	3	4	5
Elderly patient	1	2	3	4	5
Family History of Medullary Thyroid Cancer	1	2	3	4	5
Level HbA1C	1	2	3	4	5
High fasting sugar values	1	2	3	4	5
Sugar values after meals are high	1	2	3	4	5
GFR less than 15	1	2	3	4	5
History of Pancreatitis	1	2	3	4	5

The following statements relate to the perceptions of the staff regarding the treatment of diabetes in the community. You are asked to rate your agreement with the statements listed below, according to the following scale:

	Not at all	Slightly	Moderately	To a Large Extent	To a very large extent
A new diagnosis is a window of opportunity to balance diabetes and prevent the development of complications	1	2	3	4	5
It is better for the patient to be exposed to hyperglycemia than to hypoglycemia	1	2	3	4	5
	1	2	3	4	5
Treatment with two medications if the patient came to me for the first time with HBA1C over 7.5%	1	2	3	4	5
Diabetic complications must be identified,	1	2	3	4	5

otheriwse cannot be treated					
Every patient must be referred to a dietitian	1	2	3	4	5
Every patient must be referred to a nurse	1	2	3	4	5
To acheive a balanced state, patients must always start insulin and then change the treatment	1	2	3	4	5
Diabetes balance is the role of the endocrinologist	1	2	3	4	5
The level of HbA1C does not influence the choices of diabetes treatment	1	2	3	4	5
Before proceeding with treatment, the patient's adherence to treatment should be determined	1	2	3	4	5
Patient participation in the choice of treatment	1	2	3	4	5
Including a family member as a source of support for the success of the treatment	1	2	3	4	5

In this chapter you are asked to rate your consent regarding the treatment of diabetes in your clinic:

	Not at all	Slightly	Moderatel y	To a Large Extent	To a very large extent
Medication is always adjusted to the actual sugar level	1	2	3	4	5
A patient with long-acting insulin must always check fasting sugar levels	1	2	3	4	5
Long-term insulin injection is given only in the evening	1	2	3	4	5
A patient started insulin and reports that he knows the treatment, send him anyway for guidance or is not needed.	1	2	3	4	5
The level of treatment for	1	2	3	4	5

diabetics in my clinic is insufficient					
A balanced patient may consider discontinuing diabetes treatment	1	2	3	4	5
The feet of each diabetic patient must be examined at least once a year	1	2	3	4	5

Part IV. Treatment of diabetes in the community for the purpose of testing of quality of care provided by nurses:

Part B. The following statements relate to the perceptions of the nurses regarding the approach to diabetic patients, and the quality of provided medical care for diabetes. You are asked to rate your agreement with the statements listed below, according to the following scale:

Disagree	Agree to a small extent	Agree to a moderate extent	Strongly agree	Totally Agree
1	2	3	4	5

According to my impression, in my work at the clinic ...

	Not at all	Slightly	Moderatel y	To a Large Extent	To a very large extent
The patients receive a full explanation from me about the periodic tests they need to perform	1	2	3	4	5
I have a proactive approach when dealing with diabetic patients	1	2	3	4	5
Nurses dedicate enough time to talking to a diabetic patient when performing a follow-up	1	2	3	4	5
Nurses are available for patient inquiries and complaints related to diabetes,	1	2	3	4	5

even outside of working hours					
Nurses encourage the integration of family members as part of the treatment process	1	2	3	4	5
Sometimes I fail to give optimal care because of the workload	1	2	3	4	5
My care as a nurse addresses the needs of patients	1	2	3	4	5
Patients are given a comprehensive explanation of diabetes medication	1	2	3	4	5
The treatment I give in the clinic is safe and high-quality for the patient	1	2	3	4	5
Nurses encourage patients to ask questions and find out about problems or side effects in the treatment they receive	1	2	3	4	5
The quality of treatment I provide in diabetes is based on the level and quality of my knowledge in the field	1	2	3	4	5
The nurses work skillfully and professionally in diabetes	1	2	3	4	5
I contribute to improving the quality of treatment for diabetes patients	1	2	3	4	5
I regularly invite patients for follow-ups to maintain a	1	2	3	4	5

regular therapeutic sequence					
I as a nurse make sure to perform a foot estimate for each patient according to the procedure	1	2	3	4	5
To improve the quality of treatment, I seek the help of an attending physician	1	2	3	4	5
To improve the quality of treatment, I check each patient's level of responsiveness and treatment adherence, including the purchase of medication	1	2	3	4	5
To improve the quality of treatment, I refer every diabetic patient to a multi-professional team as needed as to dietitian	1	2	3	4	5

Regards.

Supporting Information S3. Supplementary Table S1. Clinical parameter changes after intervention in both the control and experimental groups.

	Control group (n = 2011)			
	Baseline	Post-intervention	Change (IC95%)	$P_{post\text{-}base}$
Weight (kg)	83.7 ± 16.8	83.2 ±16.6	0.68 (0.51-0.86)	<0.001
BMI (kg/m^2)	29.35 ± 7.06	29.11 ± 7.01	0.24 (0.03 – 0.18)	<0.001
SBP (mmHg)	132 ± 16	131 ±16	0.63 (0.05-1.21)	0.034
DBP (mmHg)	76 ± 8	76 ±8	0.12(-0.21-0.45)	0.483
Fasting Glucose (mg/dl)	133 ± 43	130 ±43	3.06 (1.58-4.54)	<0.001
HbA1c (%)	6.9 ± 1.3	6.8 ±1.3	0.77 (0.04-0.11)	<0.001
Triglycerides (mg/dl)	162 ± 152	154 ±96	8.34 (3.54-13.14)	<0.001
Total cholesterol (mg/dl)	173 ± 43	173 ±41	0.11(-1.14-1.37)	0.859
Non HDL (mg/dl)	128 ± 42	127 ±39	1.54 (0.31-2.77)	0.014
LDL (mg/dl)	97 ± 35	97 ±35	-0.53 (-1.045-0.93)	0.916
eGFR (mL/min/1.73m^2)	75.1 ± 20.5	76.3 ±21.4	-9.34 (-1.23-0.63)	<0.001

	Intervention group (n = 2088)			
	Baseline	Post-intervention	Change (IC95%)	$P_{post\text{-}base}$
Weight (kg)	85.9 ± 17.0	85.1 ±16.7	0.83(0.63-1.03)	<0.001
BMI (kg/m^2)	30.34 ± 7.16	30.05 ± 7.07	0.29(0.04 – 0.22)	<0.001
SBP (mmHg)	131 ± 16	129 ±15.4	1.21(0.64-1.79)	<0.001
DBP (mmHg)	76 ± 9	75 ± 9	0.43(0.79-0.78)	0.016
Fasting Glucose (mg/dl)	137 ± 51	127 ± 40	9.58(7.70-11.47)	<0.001
HbA1c (%)	7.2 ± 3.9	6.84 ± 1.16	0.41 (0.25-0.58)	<0.001
Triglycerides (mg/dl)	171 ± 124	164 ±140	7.14(2.38-11.90)	0.003
Total cholesterol (mg/dl)	173 ± 41	173 ± 51	0.49 (-1.22-1.32)	0.940
Non HDL (mg/dl)	131 ± 40	129 ± 41	1.53(0.3-2.75)	0.014
LDL (mg/dl)	97 ± 34	97 ± 35	0.090 (-0.91-1.09)	0.861
eGFR (mL/min/1.73m^2)	82.2 ± 20.4	83.8 ± 21.1	-1.56 (-1.89 - -1.23)	<0.001

Data represent mean ± sd. BMI: body mass index; SBP: systolic blood pressure; SBP: diastolic blood pressure. eGFR: Estimated glomerular filtration rate. Statistical differences were evaluated by a paired t test.

Supporting Information S4: R code

R Code

```
#Load Libraries

library(haven)

library(dplyr)

library(lme4)

library(lmerTest)

library(ggplot2)

library(ggeffects)


# Load SPSS file

#data <- read_sav("/ruta/al/archivo/*******************.sav")

data <- read_sav("G:/*********************************.sav")


# Check variables and types with str()

str(data)


# To ensure that categorical variables are factors and numeric variables remain

# as numbers, do the following:

data <- data %>%

  mutate_if(is.labelled, as_factor)

# Check variables again

str(data)


# Fitting the model using lmerTest

model <- lmer(var1 ~ Group + (1 | Professional), data = data)


# Get the summary with p-values

summary(model)
```

```
# Checking the model assumptions: Normality and Homoscedasticity
# Extract the residuals from the fitted model
residuales <- residuals(model)

# Histogram of the residuals
hist(residuales, main = "Histograma de los residuales", xlab = "Residuales", breaks = 30)

# QQ-plot to verify normality
qqnorm(residuales)
qqline(residuales, col = "red")

# Shapiro-Wilk normality test
shapiro.test(residuales)
#To check homocedasticity
# Plot of fitted values versus residuals
plot(fitted(modelo), residuales,
    main = "Valores ajustados vs Residuales",
    xlab = "Valores ajustados",
    ylab = "Residuales")
abline(h = 0, col = "red")

# Comparison with a null model
# Fitting the null model without the Group variable
modelo_nulo <- lmer(change_var1 ~ (1 | Professional), data = data)

# Compare the full model with the null model using ANOVA (Likelihood Ratio Test)
anova(modelo_nulo, modelo)
```
